# Suicides by pesticide ingestion in Pakistan and the impact of pesticide regulation

**DOI:** 10.1186/s12889-023-15505-1

**Published:** 2023-04-11

**Authors:** Shweta Dabholkar, Shahina Pirani, Mark Davis, Murad Khan, Michael Eddleston

**Affiliations:** 1 Centre For Pesticide Suicide Prevention, University of Edinburgh, QMRI E3.22a, 47 Little France Crescent, Edinburgh, EH16 4TJ UK; 2 Department of Psychiatry, & Brain & Mind Institute, Aga Khan University, Karachi, Pakistan; 3 Pharmacology, Toxicology & Therapeutics, University/BHF Centre for Cardiovascular Science, University of Edinburgh, Edinburgh, UK

**Keywords:** Poisoning, Organophosphate, Highly hazardous pesticides (HHP’s), Agriculture, Policy, Pesticide bans

## Abstract

**Introduction:**

Suicide is a major public health problem in Pakistan, accounting to approximately 19,331 deaths every year. Many are due to consumption of acutely toxic pesticides; however, there is a lack of national suicide data, limiting knowledge and potential for intervention. In this paper, we aimed to review the literature on pesticide self-poisoning in Pakistan to identify the most problematic pesticides in relation to national pesticide regulations.

**Methods:**

Information on the currently registered and banned pesticides was obtained from Ministry of National Food Security and Research while data on pesticide import and use was extracted from FAOSTAT. We searched the following sources for articles and research papers on poisoning in Pakistan: Cumulative Index to Nursing and Allied Health (CINAHL), Google Scholar, Applied Social Sciences Index and Abstracts (ASSIA), Excerpta Medica (EMBASE), National Library of Medicine’s MEDLINE (PUBMED), PS102YCHINFO and Pakmedinet.com using the search terms ‘self-poisoning’, ‘deliberate self-harm’, ‘suicide’, ‘methods and means of suicide’, ‘organophosphate’, ‘wheat pill’, ‘aluminium phosphide’, ‘acute poisoning’, OR ‘pesticides’, AND ‘Pakistan’.

**Results:**

As of May 2021, 382 pesticide active ingredients (substances) were registered in Pakistan, of which five were WHO hazard class Ia (extremely hazardous) and 17 WHO hazard class Ib (highly hazardous). Twenty-six pesticides, four formulations, and seven non-registered pesticides had been banned, of which two were WHO class Ia and five Ib. We identified 106 hospital-level studies of poisoning conducted in Pakistan, of which 23 did not mention self-poisoning cases and one reported no suicidal poisoning cases. We found no community or forensic medicine studies. Of 52,323 poisoning cases identified in these papers, 24,546 [47%] were due to pesticides. The most commonly identified pesticide classes were organophosphorus (OP) insecticides (13,816 cases, 56%) and the fumigant aluminium phosphide (3 g 56% tablets, often termed ‘wheat pills’; 686 cases, 2.7%). Few studies identified the particular pesticides involved or the resulting case fatality.

**Conclusion:**

We found pesticide poisoning to be a major cause of poisoning in Pakistan, with OP insecticides and the fumigant aluminium phosphide the main pesticides identified. Withdrawal of Class I pesticides (as proposed to occur nationally in 2022) and high concentration aluminium phosphide tablets should rapidly reduce suicidal deaths by reducing the case fatality for low-intention poisoning cases. National cause of death data and forensic toxicology laboratory data identifying the pesticides responsible for deaths will be important to assess impacts of the proposed national ban.

**Supplementary Information:**

The online version contains supplementary material available at 10.1186/s12889-023-15505-1.

## Introduction

Highly hazardous pesticides (HHPs) cause harmful acute or chronic health effects on people and livestock [1, 2]. They harm reproductive and neurological systems [3–6], and can lead to immunological, genotoxic, endocrine disrupting and carcinogenic conditions [7–12]. Acutely toxic HHPs have a high case fatality after consumption compared to other domestically available poisons such as overdoses of sedative or analgesic medicines [13]. At least fourteen million people have died from pesticide suicides since the Green Revolution brought these toxic chemicals into rural communities in the 1950/60s [14]. However, self-poisoning is often an impulsive decision, taken after less than 30 minutes of suicidal thoughts [15]. If a person survives the act, she/he can receive support from family and peers, reducing the risk of a repeat attempt [13–17]. If acutely toxic HHPs are made less available in these communities, a person will consume a less toxic poison, making the attempt less dangerous and increasing the chance of survival [15].

The issues of pesticide poisoning and suicide are not well described for Pakistan. National health vital statistics on suicide or poisoning are not collected, which hinders understanding of the gravity of the problem for policy level solutions. The WHO (2019) reports an estimate for the country of ~ 19,331[18] suicides per year. However, they are under-reported due to social stigma and being a criminal offence under Sect. 325 of the Pakistan Penal Code [PPC] [19, 20] until 2022. Pakistan’s progressive step in December 2022 to decriminalize suicide should improve suicide reporting. Taking into consideration the unreported suicides, it is likely that more than 19,331 suicides occur every year in Pakistan, with poisoning, firearms, and hanging being the top three means of suicide [19, 21, 22]. Pesticides are likely to be the most important means of suicide by poisoning.

In 2019, the Pakistan government’s Department of Plant Protection (Box 1) decided to phase out the use of WHO hazard class Ia (extremely hazardous [23]) and class Ib (highly hazardous) pesticides by 2022, subject to availability of alternatives [24]. This accords with WHO advice on the efficacy and cost-effectiveness of pesticide bans for suicide prevention [25, 26]. However, it is not clear whether the pesticides listed for bans are the pesticides involved in most self-harm deaths. We therefore undertook a systematic review of the issue of pesticide poisoning and suicides in Pakistan in light of the planned pesticide regulation to identify the most important pesticides [27].

**Table:** 

Text box 1. Pesticide regulation in Pakistan	
Since the formation of Pakistan in 1947, the distribution and procurement of pesticides has been administered directly by the federal government [28]. Currently, the Department of Plant Protection, under the Ministry of National Food Security and Research, monitors the import and standardization of pesticides and implements pesticide regulation. Chemical pesticides were first used in Pakistan in 1950 to deal with locusts [12]. Until 1971, pesticides were freely distributed to the farmers. The Agricultural Pesticides Ordinance of 1971 and Agricultural Pesticides Rules of 1973 were then implemented to regulate the import, manufacture, formulation and sale of registered pesticides [28, 29]. In 1980, Pakistan announced a liberalisation policy that shifted the distribution, sales and import of pesticides into the hands of the private sector [7, 30]. Essentially, liberalization facilitated the import of pesticides, complimented by a low-price structure, increasing the consumption of pesticides five-fold in just one year [7]. However, in 1983, the Pakistan Environmental Protection Ordinance included pesticide regulations due to concerns about environmental degradation [31]. The government then banned 22 pesticides from 1989 to 1993 (Table 1). A further four acutely toxic HHPs associated with many deaths in South Asia were banned between 2001 and 2012 (Table 1). However, a lack of proper enforcement of pesticide regulations [32] has enabled the continued use of some banned pesticides, such as DDT and ethylene dichloride [27, 33–35]. Pakistan has developed ‘Good Pesticide Application Practices’ for ground operations following FAO and WHO guidelines due to concerns about pesticide mishandling, misuse, and overuse [36]. It focusses on correct application practice, safety measures while applying, protecting the health of operators, transport, and storage management [36]. Pakistan has signed the Stockholm and Rotterdam Conventions. The Department of Plant Protection regularly reviews updated lists of banned chemicals from the conventions and, after approval from the Ministry of National Food Security and Research, places a ban on such chemicals.	

**Table 1:** **Pesticides banned in Pakistan by 2021** [36, 182, 183] (All except dichlorvos and ethylene dibromide were banned for being harmful to human health and environment health; no reasons were given for these two pesticides)

Pesticides	Class		Year	Form
BHC (benzene hexachloride)	II		1992	Active Ingredients
Binapacryl	O		1994	Active Ingredients
Bromophos Ethyl	II		1994	Active Ingredients
Captafol	Ia		1994	Active Ingredients
Chlordimeform	O		1994	Active Ingredients
Chlorobenzilate	O		1994	Active Ingredients
Chlorthiophos	O		1994	Active Ingredients
Cyhexatin	II		1994	Active Ingredients
Dalapon	U		1994	Active Ingredients
DDT (Dichlorodiphenyltrichloroethane)	II		1994	Active Ingredients
Dibromochloropropane + Dibromochloropropene	O		1994	Active Ingredients
Dicrotophos	Ib		1994	Active Ingredients
Dieldrin	O		1994	Active Ingredients
Disulfuton	Ia		1994	Active Ingredients
Endrin	O		1994	Active Ingredients
Ethylene dichloride + Carbontetrachloride	FM		1994	Active Ingredients
Leptophos	O		1994	Active Ingredients
Mercury compounds	Ia, Ib, O		1994	Active Ingredients
Mevinphos	Ia		1994	Active Ingredients
Toxaphene	O		1994	Active Ingredients
Zineb	U		1994	Active Ingredients
Heptachlor	0		1997	Active Ingredients
Methyl Parathion	Ib		2001	Active Ingredients
Monocrotophos	Ib		2005	Formulation
Methamidophos	Ib		2001	Formulation
Endosulfan	II		2012	Active Ingredients
Phosphamidon (above 500 g/l)	Ia		NA	Formulation
Dichlorvos (above 500 g/l)	Ib		NA	Formulation
Aldrin (POP/PIC)	O		NA	Not registered pesticide
Mirex (POP)	O		NA	Not registered pesticide
Chlordane (POP/PIC)	O		NA	Not registered pesticide
Dinoseb (PIC)	O		NA	Not registered pesticide
Ethylene di bromide (PIC)	FM		NA	Not registered pesticide
Parathion (PIC)	Ia		NA	Not registered pesticide
Fluoroacetate (PIC)	Ia		NA	Not registered pesticide
Monocrotophos (above 400 g/l)	Ib		NA	Formulation
Methamidophos (above 600 g/l)	Ib		NA	Formulation

## Methods

### Pesticide regulation

Information on pesticide regulation was obtained from the 2018 Pesticide Handbook published by the Pakistan Agricultural Research Council, Ministry of National Food Security and Research [36].

### Pesticide consumption

Data on pesticide consumption and national level per hectare pesticide consumption in Pakistan were obtained from FAOSTAT. However, Pakistan has not reported pesticide use per hectare to the FAO since 2013; compound specific consumption data are not available.

### Pesticide poisoning and suicides

The review was conducted using Arksey and O’Malley’s (2005)[37] framework, that described five stages: identifying the research question, identifying relevant studies, study selection, charting the data and summarizing and reporting the results. The methods of this review described in the light of above stages are as follows:-

Stage 1: The review was conducted to identify the poisoning cases as a method of suicide or self-harm in Pakistan.

Stage 2: In order to identify and include relevant studies, we used combination of key terms including ‘self-poisoning’, ‘deliberate self-harm’, ‘suicide’, ‘methods and means of suicide’, ‘organophosphate’, ‘wheat pill’, ‘aluminium phosphide’, ‘acute poisoning’, or ‘pesticides’, AND ‘Pakistan’. We searched different electronic databases including Cumulative Index to Nursing and Allied Health (CINAHL), Google Scholar, Applied Social Sciences Index and Abstracts (ASSIA), Excerpta Medica (EMBASE), National Library of Medicine’s MEDLINE (PUBMED), and PSYCHINFO. Pakmedinet.com, a Pakistani medical literature search website, was also searched for relevant literature. These databases were searched from their beginning until June 2021. In addition, an ancestry approach was taken in which the reference lists of retrieved articles were hand checked for relevant references.

The selected studies were assessed against the following inclusion criteria:

(i) Studies conducted on suicide or self-harm;
(ii) Studies mentioned poisoning as a method of suicide or self-harm;
(iii) Suicidal behaviour in both genders and across all ages;
(iv) English as the publication language;
(v) Studies used retrospective, cross-sectional, or prospective study designs.
(vi) Studies conducted within the geographical boundaries of Pakistan.

On the other hand, any study that did not fulfill the inclusion criteria and those conducted on Pakistani people residing outside the country were excluded from this review.

Stage 3: A two stage study selection process was followed. In the initial phase, titles and abstracts of the study were screened. In the second stage, full text of the articles was retrieved after applying the eligibility criteria.

Stage 4: Based on literature review and study objectives, the data extraction form was developed in consultation with the subject expert. This form included information on study design, time of study, year, place of study, number of suicidal cases, number of poisoning cases, type of poisoning, age, gender, outcome, and mortality due to suicidal poisoning. The data was entered in Microsoft Excel. The form was pilot tested, and modifications were made accordingly.

Stage 5: Information on pesticide class, number of pesticides poisonings, number of suicidal pesticides poisoning, gender, age and mortality due to suicidal poisoning were the basis of analysis. Throughout the process, team meetings were held, and each step was discussed.

### Statistical analysis

Simple descriptive statistics were used to analyse the data.

## Results

### Pesticides registered in Pakistan

As of April 2022, 382 pesticide active ingredients (substances) were registered in Pakistan of which five are WHO hazard class Ia (extremely hazardous), 17 class Ib (highly hazardous), and 36 class II (moderately hazardous) (Table 2; online supplementary table) [Table S1- S10 series].

**Table 2:** Registered pesticides in Pakistan by their type [36]

Type of pesticide (group name)	Number registered
INSECTICIDES	
Organophosphorus insecticides	34
Organophosphorus combination products	13
Carbamate insecticides	13
Carbamate combination products	2
Pyrethroid insecticides	16
Pyrethroid combination products*	33
Neonicotinoids	25
Miscellaneous insecticides	56
Homeopathic insecticides	1
Fungicides	61
Herbicides	111
Acaricides	11
Rodenticide	2
Fumigants	3
Nematicides	1

* Excludes the combination products with OP or carbamate insecticides

Twenty-six pesticides, four formulations, and seven non-registered pesticides are currently banned in Pakistan (Table 1). Four of these pesticides are WHO hazard class Ia, four class Ib, and five class II; two were classified as unlikely to present acute hazard. The Department of Plant Protection announced in 2019 that all the currently registered WHO class I pesticides (five class Ia, 17 class Ib) would be withdrawn in 2022 [24].

### Use of pesticides in Pakistan

The annual consumption of pesticides in Pakistan is 130,000 metric tonnes, of which 90% is applied on cotton, rice, vegetables and fruits [38]; China is the source of 91% pesticides in Pakistan [39]. The pesticide market was valued at USD 220 million in 2019 and is forecast to reach USD 349.5 million by 2025 [40]. Use of biopesticides is low due to lack of expertise in manufacture and use [39]. The Pakistan pesticide sector mainly consists of 272 small scale importers that sell the products through dealers to end users [39].

Pakistan uses 69% of its pesticides on cotton [7], so any change in the area or technology of cotton production impacts national consumption of pesticides[41]. Thus, the decreasing pesticide use in Pakistan is associated with a reducing area of cotton agriculture and introduction of Bt. cotton in 2002 [27]. Relative to neighbouring countries, Pakistan uses a very low quantity. In 2012, China, India and Pakistan used 13.35 kg/hectare, 0.31 kg/hectare, and 0.03 kg/hectare pesticides on arable land, respectively. Pesticide consumption data post 2013 is not available (due to lack of reporting from Pakistan).

### Pesticide self-poisoning in Pakistan

We identified a total of 106 hospital studies [41–146], starting from 1977, from across Pakistan, of which 83 mentioned the intention of poisoning. We did not identify any community level studies or forensic medicine studies reporting fatal poisoning cases.

**Fig. 1:**
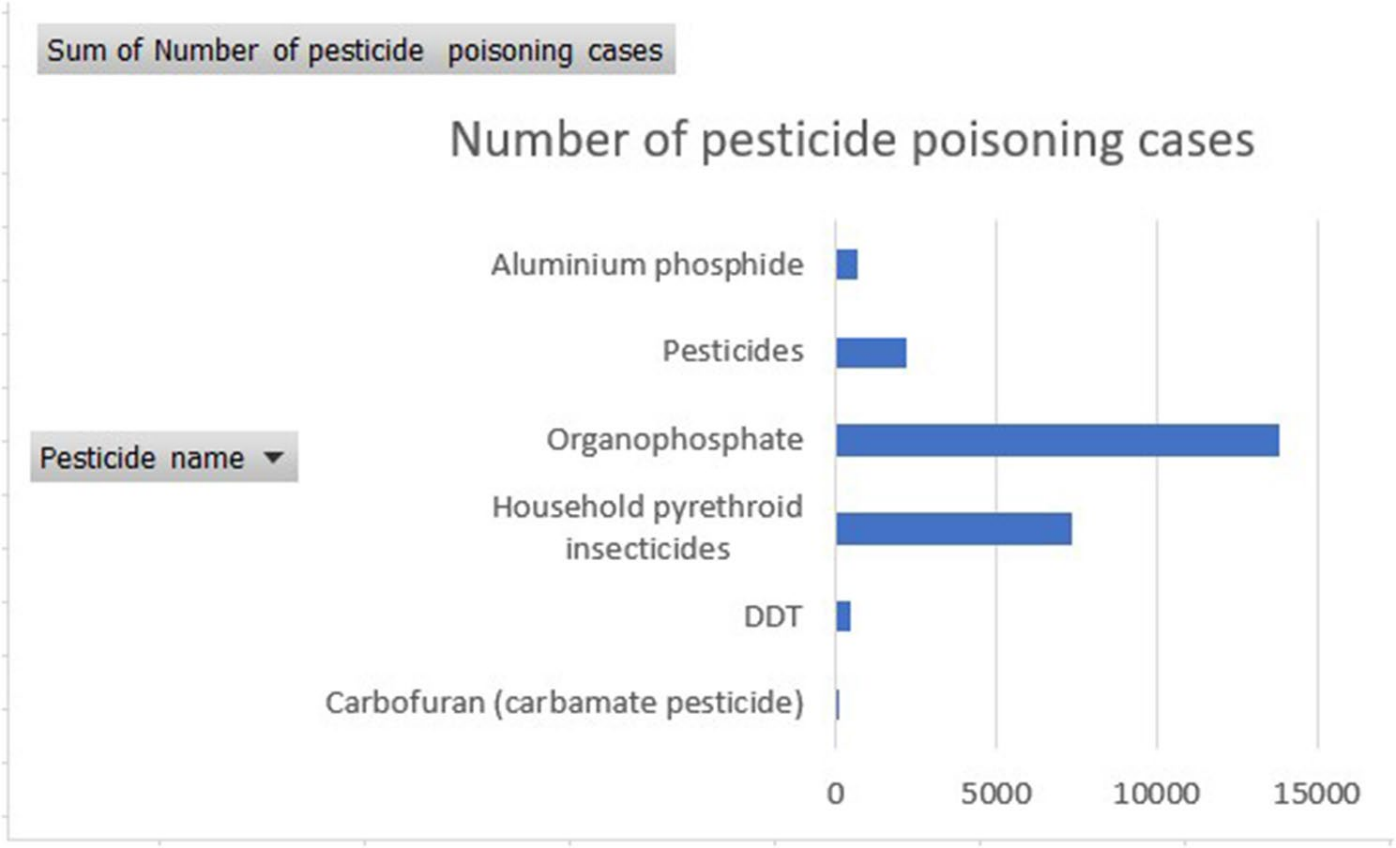
Number of pesticide poisoning cases

**Fig. 2:**
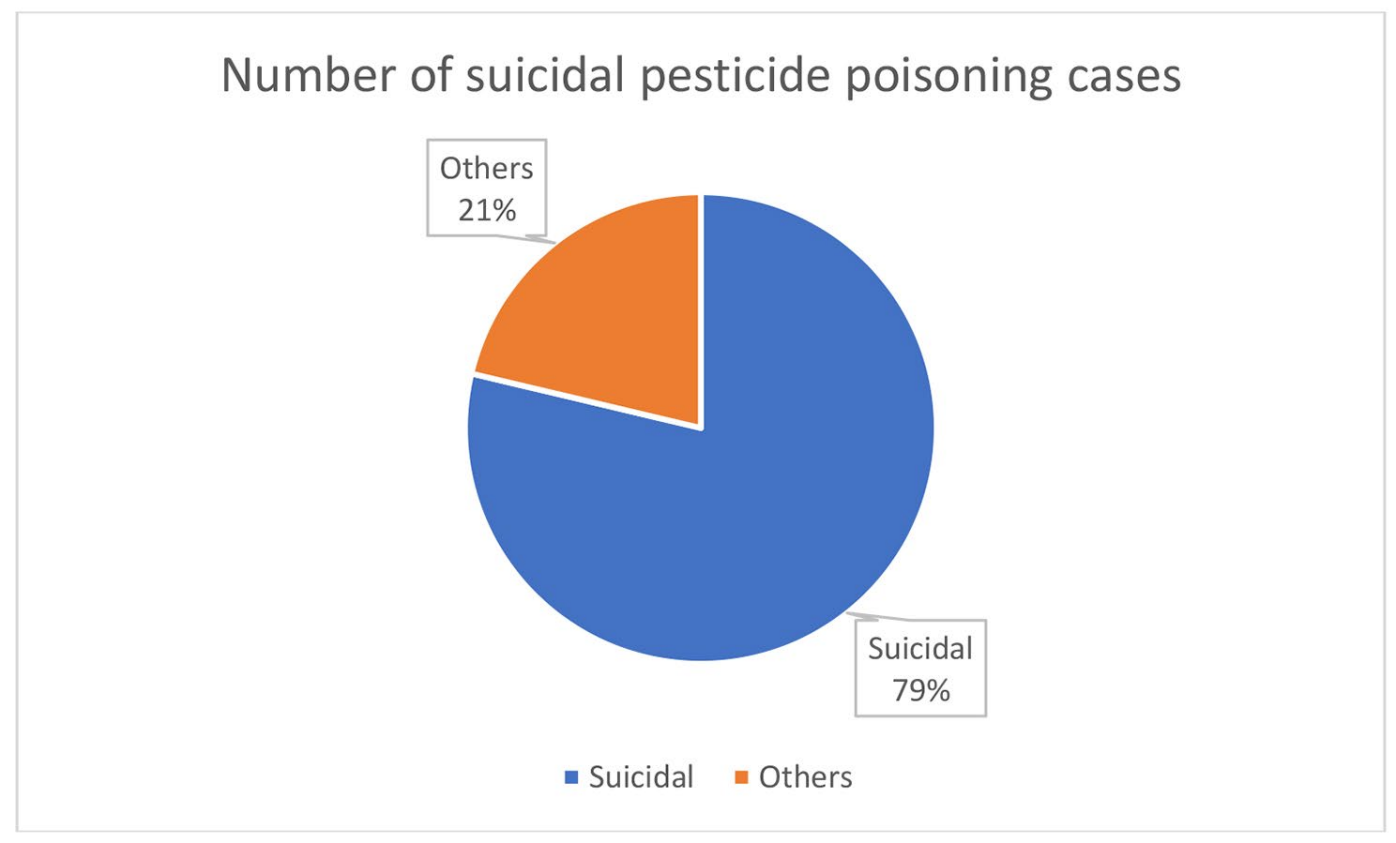
Suicidal Pesticides poisoning cases

The papers reported a total of 53,323 cases of poisoning, of which 24,546 [46.0%] were due to pesticides [Fig. 1]. OP insecticides were responsible for 13,816 (56.2%) of the total pesticide poisoning cases; unfortunately, no data were presented on the identity of the most important OP insecticides for poisoning or deaths. Two studies mentioned malathion and parathion as being commonly used OP pesticides [91, 134]; remarkably just two studies reported pesticide specific poisoning data: in two case series, 48 (23%) cases were due to dichlorvos [136] and 59 (46%) cases due to paraquat [44]. The fumigant aluminium phosphide - as a 3 g 56% tablet - was identified as being responsible for 686 (3%) poisoning cases. OP insecticide self-poisoning was first reported in 1977 [147] while aluminium phosphide self-poisoning was first reported in 1997 [126]. A single paper, reporting 11925 cases admitted to 3 hospitals in Karachi after self-poisoning in 2006-11, noted that household pyrethroid insecticide mixtures were responsible for 62% of cases [68]. Among all pesticide poisoning papers, 79% of cases were due to self-poisoning [Fig. 2].

The data did not allow the number of deaths from pesticide poisoning to be calculated, because deaths were often not reported and, if reported, pesticide deaths were usually mixed in with other poisons. However, estimates could be generated from studies that reported only pesticide poisoning and the number of deaths. Twenty-one studies of OP insecticide poisoning reported 376/3504 deaths (13% case fatality) while six studies of aluminium phosphide poisoning reported 163/262 deaths (62% case fatality).

Data on gender were available in 74 papers reporting pesticide poisoning only. In these studies, 44% and 49% of cases were male and female (7% unknown), respectively. The 25 studies that reported only OP poisoning and gender data included 44% males and 48% female. Similarly, the seven studies that reported only aluminium phosphide poisoning included 47% males and 52% females. There were no data on the number of deaths by gender in these studies.

Other types of poisoning identified from our literature review included overdose of medicines, drugs, and household compounds. Of note, paraphenylenediamine (PPD) hair dye was reported in 20 publications and responsible for many self-poisoning cases and some deaths [49, 50, 62, 69, 70, 75, 76, 79, 80, 86, 88, 92, 104, 105, 122, 124, 129, 142]. There was a more marked female/male imbalance in these cases (2862 women vs. 1176 men).

## Discussion

Our systematic literature review found the continued use of acutely toxic HHPs in Pakistani agriculture and the importance of OP insecticides and aluminium phosphide tablets for self-poisoning in Pakistan. Unfortunately, there was a remarkable lack of data on the number of deaths specific to pesticide poisoning, gender, and identity of pesticides responsible for most deaths. Experience from other countries [148, 149] suggest that acutely toxic OP insecticides and aluminium phosphide are likely to be responsible for many deaths.

OP insecticides inhibit the enzyme acetylcholinesterase (AChE) resulting in accumulation of acetylcholine and overstimulation of nervous system receptors [2, 150, 151]. Deaths occur from respiratory arrest, particularly if patients cannot be rapidly and safely transferred to hospital [150]. A Karachi-based hospital study showed that 46.1% of the 2,546 admitted poisoning cases were due to OP insecticides, with the highest case fatality of any poison (3.6%)[46]. This is consistent with their wide use across South Asia for agriculture and hence for self-poisoning [148]. However, the urban location of this hospital and relatively low case fatality [152, 153] suggest that many of the patients admitted to this hospital are ‘survivors’, with most severely poisoned patients not surviving to arrive at this specialist unit or being treated in rural district hospitals (not this specialist centre).

Aluminium phosphide or ‘wheat pills’ are widely used as a fumigant and rodenticide for stored rice and wheat, the major crops grown in Pakistan. It is extremely toxic, releasing phosphine gas following exposure to moisture (including exposure to air or following ingestion) [154]. Case fatality amongst those who ingest aluminium phosphide tablets is greater than 50% despite treatment [155–159]. Wheat pill poisoning is common in north Pakistani wheat-growing areas such as Peshawar [48], Lahore [109], Rawalpindi and Sahiwal [145], while such poisonings have not been noted in the southern Sindh region where wheat is not a key crop [21], showing again the importance of agent availability for self-poisoning. Similarly, aluminium phosphide tablets are a major problem in north India but not the south where they are little used in agriculture [156].

Many less toxic pesticides, other than OP insecticides and aluminium phosphide, are used in agriculture and thus available for self-harm in rural Asia. Unfortunately the literature available from Pakistan does not allow these pesticides to be identified, as has been done for example in Sri Lanka [160]. Poisoning with most of these less toxic pesticides can be successfully treated as long as patients do not aspirate their stomach contents [150]. Deaths are much less common after self-poisoning with these pesticides [153], emphasising the importance of banning acutely toxic HHPs to reduce pesticide poisoning deaths by reducing the case fatality [161, 162].

In 2019, the Pakistan government proposed to ban all 22 WHO class Ia and Ib hazardous pesticides still in agricultural use by 2022, if alternatives could be identified [24]. Such bans of acutely toxic HHPs should reduce deaths from suicidal and occupational pesticide poisoning [163]. However, since aluminium phosphide has not been classified by the WHO [23], such a regulation will not remove this pesticide, which is defined as highly hazardous by the FAO/WHO Joint Meeting on Pesticide Management (JMPM)’s criterion 8 [2] and kills many young people in Pakistan [64, 66, 78, 98, 109]. Replacement of the 3 g 56% tablets with a low concentration granular formulation should reduce deaths, as has been seen in India [164, 165] and recently implemented in Nepal [166, 167].

Pesticides are used in agriculture to prevent pest attacks and protect crops. Pest and locust attacks are common in Pakistan, so the use of pesticides is considered important for agricultural productivity [168]. However, Pakistan’s neighbouring countries, Sri Lanka [169] Bangladesh [170] and Nepal [167], have implemented similar bans without affecting their agricultural productivity by introducing suitable safer alternatives [171]. Pakistan could benefit from the experiences of these South Asian neighbours.

Accessibility to highly lethal means for suicide is a key factor for determining suicide rates. For example, suicides by using guns is common in gun-carrying nations [172, 173], carbon monoxide self-poisoning has become common in East Asian countries where charcoal is available over the counter [174, 175], and drowning is specific to nations like Netherlands with many bodies of water [176] [177]. Two recent review articles on suicide in Pakistan [18][20] show poisoning to be the second most common mode of suicides [19] and the agents to be specific to regions where they are easily accessible [21]. They review the different methods of self-harm [19] and compare the incidence of suicides in rural and urban areas of Pakistan [21]. While OP insecticide poisoning was common in South Punjab and central Sindh, aluminium phosphide poisoning was higher in North Punjab [21]. Paraphenylenediamine was a commonly used agent in South Punjab and household chemical agents like rat poison were commonly used in in urban areas [21].

**Fig. 3:**
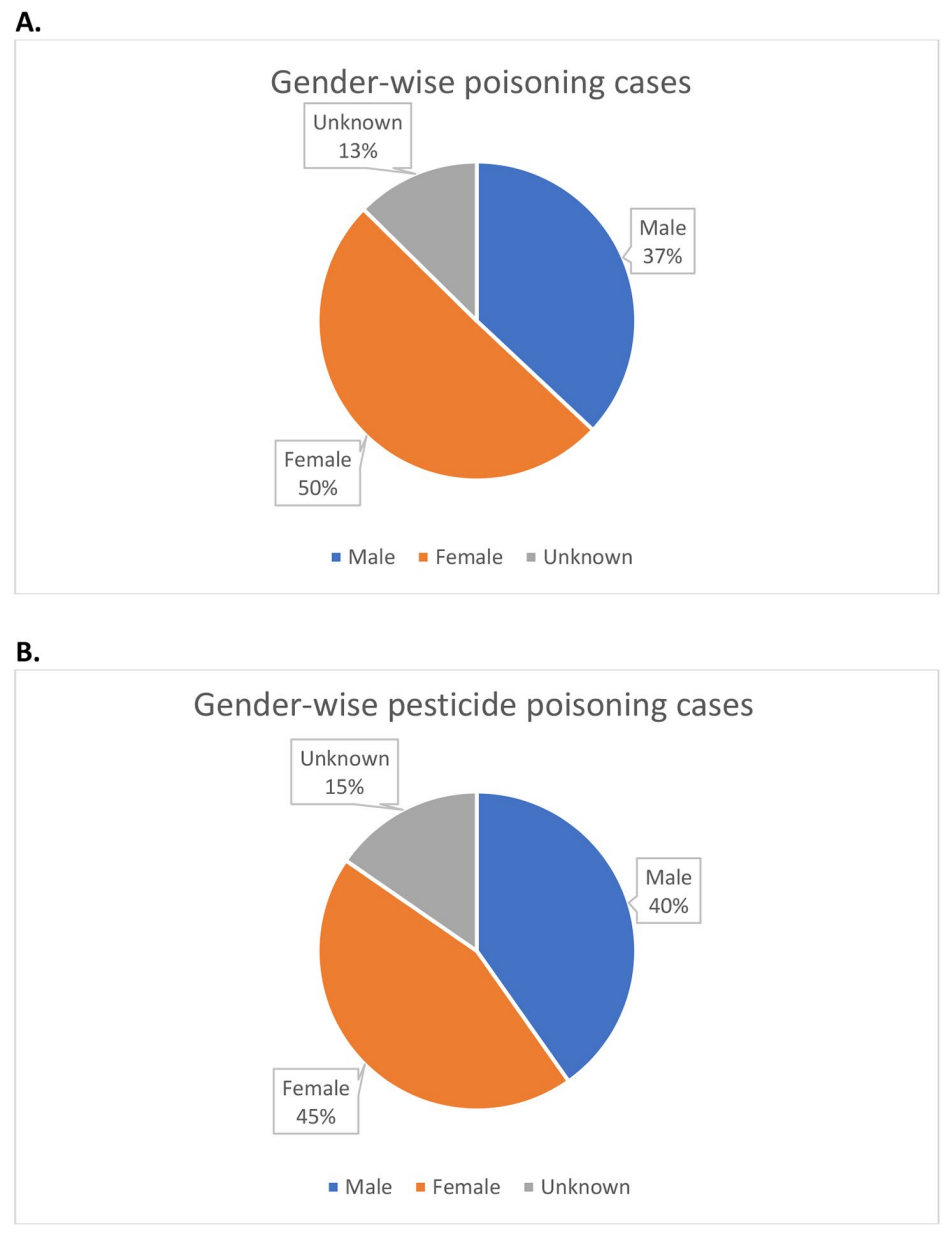
A. All poisoning cases and B. pesticide poisoning cases [only including the 33 studies that reported 100% pesticide poisoning] by gender

Self-poisoning is most common amongst individuals below the age of 30 [178] and females [21] [Fig. 3], while the case fatality is highest amongst males. Mental health awareness campaigns, provision of resources and staff training for prompt treatment of poisoning cases, establishment of a central data collection system to diagnose and investigate suicides, and stricter pesticide regulations are key recommendations of both reviews [21, 178].

There are issues with data collection for poisoning and suicide in Pakistan. Poisoning cases, including pesticide self-poisoning cases, are recorded under the broad category of injuries in Pakistan by emergency departments (ED) of hospitals and/or the police. There is a difference in the number and type of cases reported by both sources [20]. Violent or fatal cases are usually reported by the police while injury cases or self-harm are more reported by the EDs [20]. However, there is no centralized system- either at provincial or national levels- to collect suicidal or injury data, resulting in poor quality data for government policy decisions.

We found more female pesticide poisoning cases in the literature than male cases, consistent with data from other countries. However, despite the lack of high-quality data, more men than women are believed to die from pesticide poisoning [21]. More research is required to understand the true rates of self-harm by gender in Pakistan and whether there any gender-specific reasons for self-harm exist.

None of the cited papers differentiated between urban and rural cases. As all the studies were hospital based, it is likely that many cases were derived from urban areas, with cases from rural areas being under-represented. Information on the use of agricultural pesticides in self-poisoning is likely limited compared to insecticides that are available in urban areas.

### Limitations

Lack of official national data on suicides by means (and poison) restricted our research to published poisoning studies. We were able to find the most common pesticide classes associated with fatal self-poisoning, based on our review of 106 hospital studies. Unfortunately, these studies do not identify the specific pesticides that caused lethal poisoning (unless all reported poisoning cases were due to a single agent). Identification of the type of compound consumed by an individual at hospital level is important to understand the chemicals responsible and allow evidence-based policy decisions. We were unable to find any community or forensic medicine studies that might have provided some information on the number of people who died before hospital presentation, or died in different level hospitals, or the compounds involved.

The Pakistan government has previously banned pesticides due to their detrimental impact on human and environmental health. However, the lack of national monitoring and data collection on suicides make it difficult to measure the impact of these bans.

## Conclusion

OP insecticides and aluminum phosphide fumigant tablets are the two most important pesticides used in self-poisoning and general poisoning cases in Pakistan. The proposed 2022 ban on HHPs would legally restrict the use of the most toxic OP insecticides, but not the use of high concentration aluminium phosphide tablets. Hence, Pakistan needs to devise an exclusive strategy for eliminating the risk of aluminium phosphide tablets or include it in the list of bans. Most pesticide poisoning cases are impulsive decisions, a response to acute stress [164, 179]. Removal of acutely toxic HHPs from agriculture will allow the self-harm impulse to pass without exposure to a lethal means, increasing the chance of survival [180, 181] as has clearly been shown in Sri Lanka [171]. There is an urgent need to set up effective data collection for suicide and poisoning in Pakistan for policy purposes.

## Electronic supplementary material

Below is the link to the electronic supplementary material.

Supplementary Material 1

Supplementary Material 2

## Data Availability

All data generated or analysed during this study are included in this published article and its supplementary information files. We conducted a systematic review of self-poisoning studies in Pakistan from 1977. Our data sources and research methods are described in line 149 under the title of pesticide poisoning and suicides. All authors declare no competing interest.
