# Supplementary material for: Suicides by pesticide ingestion in Pakistan and the impact of pesticide regulation
Source: BMC Public Health. 2023 Apr 11;23:676. doi: 10.1186/s12889-023-15505-1 (PMC10088141; doi:10.1186/s12889-023-15505-1)
Supplement: Supplementary file 2 — Supplementary Material 2 [file 12889_2023_15505_MOESM2_ESM.docx]

**Online data**

**Table S series: Online supplementary table: WHO Hazard Class Ia, Ib and II pesticides registered in Pakistan (2021)**

**Table S1: Organophosphorus pesticides registered in Pakistan.**

|  | **Compound** | **Class** |
| --- | --- | --- |
| 1 | Acephate | II |
| 2 | Azinphos Methyl | Ib |
| 3 | Bromophos/Bromofos | II |
| 4 | Chlorfenvinphos | Ib |
| 5 | Chlorpyrifos | II |
| 7 | Diazinon | II |
| 8 | Dichlorvos | Ib |
| 9 | Dimethoate | II |
| 10 | Ethoprophos | Ia |
| 11 | Fenitrothion | II |
| 12 | Fenthion | II |
| 13 | Formothion | II |
| 14 | Isothioate | O |
| 15 | Isoxathion | Ib |
| 16 | Isazofos | O |
| 18 | Mephosfolan | O |
| 19 | Methacrifos | II |
| 20 | Methidathion | Ib |
| 21 | Oxydemeton methyl | Ib |
| 22 | Phenthoate | II |
| 23 | Phorate | Ia |
| 24 | Phosalone | II |
| 25 | Phosphamidon | Ia |
| 26 | Pirimiphos ethyl | NA |
| 27 | Pirimiphos methyl | NA |
| 28 | Profenofos | II |
| 29 | Pyridaphenthion | II |
| 30 | Quinalphos | II |
| 31 | Thiofanox | Ib |
| 32 | Triazophos | Ib |
| 33 | Trichlorphon | II |
| 34 | Vamidothion | Ib |

**Table S2. Pyrethroid insecticides registered in Pakistan.**

|  |  | **Compound** | **Class** |
| --- | --- | --- | --- |
| 1 |  | Alphamethrin/ Alpha-cypermethrin | NA |
| 2 |  | Bifenthrin | II |
| 3 |  | Betacyfluthrin | NA |
| 4 |  | Betacypermethrin | NA |
| 5 |  | Cyfluthrin | Ib |
| 6 |  | Cyhalothrin/ Lambda Cyhalothrin | II |
| 7 |  | Cypermethrin | II |
| 8 |  | Decamethrin/ Deltamethrin | II |
| 9 |  | Esfenvalerate | II |
| 10 |  | Fenpropathrin | II |
| 11 |  | Fenvalerate | II |
| 12 |  | Fluvalinate | II |
| 13 |  | Flucythrinate | Ib |
| 14 |  | Gamma-cyhalothrin | NA |
| 15 |  | Lambda Cyhalothrin | II |
| 16 |  | Permethrin | II |
| 17 |  | Tralomethrin | II |

**Table S3. Neonicotinoid insecticides registered in Pakistan**

|  | **Compound** | **Class** |
| --- | --- | --- |
| 1 | Abamectin + Imidacloprid | Ib |
| 2 | Abamectin + Thiamethoxam | Ib |
| 3 | Acetamiprid | II |
| 4 | Fipronil + Imidacloprid | II |
| 5 | Imidacloprid | II |
| 6 | Imidacloprid 20% + acetamiprid 0.2% | II |
| 7 | Imidacloprid 210 G/L + Beta Cyfluthrin 90 G/L | Ib |
| 8 | Imidacloprid + Fipronil | II |
| 9 | Imidachloprid + Monomehypo 70 + 02% | II |
| 10 | Imidachlorpid+36% + Tebuconazole 1.25% | II |
| 11 | Imidachloprid 180 gm/lit + Tebuconazole 6.25 gm/lit | II |
| 12 | Imidacloprid 360 gm/lit + Tebuconazole 12.5 gm/lit | II |
| 13 | Pyriproxyfen+ Imidacloprid | II |
| 14 | Spirotetramat 12% + Imidacloprid 36% | II |
| 15 | Spirotetramat 120 g/l + Imidacloprid 260 g/l | II |
| 16 | Abamectin+ Nitenpyram | Ib |
| 17 | Abamectin+ Thiamethoxam | Ib |
| 18 | Buprofezin+ Nitenpyram | II |
| 19 | Clothianidin | II |
| 20 | Clothianidin + Methiocarb | Ib |
| 21 | Nitenpyram | II |
| 22 | Nitenpyram + Chlorfenapyr | II |
| 23 | Nitenpyram + Pymetrozine | II |
| 24 | Thiacloprid | II |
| 25 | Thiamethoxam | II |
| 26 | Thiamethoxam 200 gai+ Chlorantraniliprole 100 gai/L | II |
| 27 | Thiamethoxam + Chlorantraniliprole | II |

**Table S4. Carbamate insecticides registered in Pakistan.**

| **Sr.no** | **Compound** | **Class** |
| --- | --- | --- |
| 1 | Aldicarb | Ia |
| 2 | Benfuracarb | II |
| 3 | Fenobucarb (B.P.M.C.) | NA |
| 4 | Carbaryl | II |
| 5 | Carbofuran | Ib |
| 6 | Carbosulfan | II |
| 7 | Cartap Hydrochloride | II |
| 8 | Furathiocarb | Ib |
| 9 | Isoprocarb | II |
| 10 | Methomyl | Ib |
| 11 | Metolcarb | II |
| 12 | Pirimicarb | NA |
| 13 | Thiodicarb | II |

**Table S5. Fungicides registered in Pakistan**

| **Sr.no** | **Compound** | **Class** |
| --- | --- | --- |
| 1 | Copper oxychloride | II |
| 2 | Difenoconazole | II |
| 3 | Difenoconazole + cyproconazole | II |
| 4 | Difenoconazole + propiconazole | II |
| 5 | Diniconazole | II |
| 6 | Metalaxyl | II |
| 7 | Myclobutanil | II |
| 8 | Oxadixyl + mancozeb | II |
| 9 | Propiconazole | II |
| 10 | Pyrazophos | II |
| 11 | Tebuconazole | II |
| 12 | Tebuconazole + Trifloxystrobin | II |
| 13 | Triadimenol | II |
| 14 | Triadimefon | II |
| 15 | Tricyclazole | II |
| 16 | Tridemorph | II |

**Table S6. Herbicides registered in Pakistan**

| **Sr.no** | **Compound** | **Class** |
| --- | --- | --- |
| 1 | Acetachlor + Pendimethalin | II |
| 2 | Bentazon | II |
| 3 | Bromoxynil + MCPA | II |
| 4 | Bromoxynil + MCPA + Tribenuron methyl | II |
| 5 | Bromoxynil + Ametryn | II |
| 6 | Bromoxynil Octonoate + Heptanoate + MCPA | II |
| 7 | Bromoxynil Actanoate + MCPA Isostylester | II |
| 8 | Butralin | II |
| 9 | Carfentra-zone Ethyl + IPU Isoproturon | II |
| 10 | Clomazone | II |
| 11 | Clomazone Pendimethalin | II |
| 12 | Fenoxaprop 8% + Metribuzin 8% | II |
| 13 | Glufosinate Ammonium | II |
| 14 | Haloxyfop-r-Methyl | II |
| 15 | Haloxyfop-p-Ethyl | II |
| 16 | Isoproturon | II |
| 17 | Isoproturon+Bensulfuron ethyl | II |
| 18 | Isoproturon +Bromoxynil + MCPA | II |
| 19 | Isoproturon + Diflufonican | II |
| 20 | Isoxaflutole + Atrazine 50 + 500 | III |
| 21 | Metolachlor+ Pendimethalin | II |
| 22 | Metribuzin | II |
| 23 | Metribuzin + Clomazone | II |
| 24 | Molinate | II |
| 25 | Oxadixyl | II |
| 26 | Paraquat | II |
| 27 | Pendimethalin | II |
| 28 | Pendimethalin + Prometryn | II |
| 29 | Propanil | II |
| 30 | Pyrazosulfuron | II |
| 31 | Tebuthiuron | II |
| 32 | Trifluralin + 2, 4-D | II |
| 33 | Triflollxysulfuron Sodium + Ametryn. | II |

**Table S7. Acaricides registered in Pakistan.**

| **Sr.no** | **Compound** | **Class** |
| --- | --- | --- |
| 1 | Amitraz | II |
| 2 | Azocyclotin | II |
| 3 | Dicofol | II |
| 4 | Ethion | II |
| 5 | Fenpyroximate | II |
| 6 | Pyridaben | II |

**Table S8. Rodenticides registered in Pakistan**

| **Sr.no** | **Compound** | **Class** |
| --- | --- | --- |
| 1 | Brodifacoum Burrow | Ia |
| 2 | Zinc Phosphide | Ib |

**Table S9. Fumigants registered in Pakistan**

| **Sr.no** | **Compound** | **Class** |
| --- | --- | --- |
| 1 | Aluminium Phosphide | FM |
| 2 | Magnesium Phosphide | FM |

**Table S10. Nematicides registered in Pakistan**

| **Sr.no** | **Compound** | **Class** |
| --- | --- | --- |
| 1 | Cadusafos | Ib |
